# Genetic diversity and epidemiology of accessory gene regulator loci in *Clostridioides difficile*

**DOI:** 10.1099/acmi.0.000134

**Published:** 2020-05-14

**Authors:** Yuta Okada, Shu Okugawa, Mahoko Ikeda, Tatsuya Kobayashi, Ryoichi Saito, Yoshimi Higurashi, Kyoji Moriya

**Affiliations:** ^1^​ Department of Infectious Diseases, The University of Tokyo Hospital, Bunkyo-ku, Tokyo, Japan; ^2^​ Department of Infection Control and Prevention, The University of Tokyo Hospital, Bunkyo-ku, Tokyo, Japan; ^3^​ Department of Molecular Microbiology, Graduate School of Medical and Dental Sciences, Tokyo Medical and Dental University, Bunkyo-ku, Tokyo, Japan

**Keywords:** accessory gene regulator, *Clostridioides difficile*, multilocus sequence typing, phylogenetic analysis, quorum sensing

## Abstract

Quorum sensing is known to regulate bacterial virulence, and the accessory gene regulator (*agr*) loci is one of the genetic loci responsible for its regulation. Recent reports examining *Clostridioides difficile* show that two *agr* loci, *agr1* and *agr2,* regulate toxin production, but the diversity of *agr* loci and their epidemiology is unknown. In our study, *in silico* analysis was performed to research genetic diversity of *agr*, and *C. difficile* isolates from clinical samples underwent multilocus sequence typing (MLST) and PCR analysis of *agr* loci. To reveal the distribution of *agr* among different strains, phylogenetic analysis was also performed. In our *in silico* analysis, two different subtypes, named *agr2R* and *agr2M*, were found in *agr2,* which were previously reported. PCR analysis of 133 *C *. *difficile* isolates showed that 131 strains had *agr1*, 61 strains had *agr2R*, and 26 strains had *agr2M; agr2R* was mainly found in clade 1 or clade 2 organisms, whereas *agr2M* was only found in clade 4. With rare exception, *agr1*-negative sequence types (STs) belonged to clade C-Ⅰ and C-Ⅲ, and one clade 4 strain had *agr2R*. Our study revealed subtypes of *agr2* not previously recognized, and the distribution of several *agr* loci in *C. difficile *. These findings provide a foundation for further functional and clinical research of the *agr* loci.

## Data Summary

The genomic sequences of ‘*agrD’* and ‘*agrB’* on the whole-genome sequence of *C. difficile* strain 630 (GenBank accession number: AM180355.1) and the sequences of CDR20291_3187, CDR20291_3187A, CDR20291_3188, CDR20291_3189 on the whole-genome sequence of R20291(GenBank accession number: FN545816.1) were used as templates to search for similar genetic sequences on the *C. difficile* whole-genome sequence registered in the nucleotide blast (https://blast.ncbi.nlm.nih.gov/Blast.cgi) database.

## Introduction

*Clostridioides difficile* is an obligate anaerobic bacterium with the ability to form spores. First described in 1978 as a clinical entity, *C. difficile* infection is often characterized by gastrointestinal symptoms like diarrhea. Moreover, potentially lethal manifestations of this disease, such as toxic megacolon or sepsis, are sometimes observed [1, 2]. More than 40 years have passed since *C. difficile* infection was first reported, and now toxin A and B produced by *C. difficile* are known to be the culprit of this usually nosocomial disease [2]. However, the mechanism regulating the production and extracellular release of these toxins is not fully understood, and knowledge leading to development of novel drugs is of importance.

Bacterial quorum sensing is a topic of rapidly expanding interest. Quorum sensing refers to dynamic control of bacterial density, metabolism and various physiological activities mediated by signalling molecules that the bacteria themselves produce [3]. As the signalling molecule accumulates in the surrounding environment, bacteria sense the increase of cell density and thus may change their collective behaviour accordingly. In some bacterial species, a relationship between quorum sensing and specific virulence factors has been observed [4–6].

The *agr* system is one such quorum-sensing system in bacteria that has been described in the literature. A few reports show that dysfunction of the *agr* system leads to decreased production and activity of *C. difficile* toxin production [7–9]. The *agr* system and its orthologues are known to be present across firmicutes including *Staphylococcus aureus* [10]. In *S. aureus *, links between many virulence factors and the *agr* system are reported, and such associations are also investigated in other organisms possessing genes akin to staphylococcal *agr* [11, 12].

In contrast, in *C. difficile *, the presence of strains only having *agrB* and *agrD (agr1* locus) and strains that additionally have a complete set of four *agr* genes, *agrC*, *agrA*, *agrB* and *agrD* (traditionally called the *agr2* locus), was previously reported in clinical samples. It was suggested that most strains fall into the latter group [13]. However, the genetic diversity of the *agr* loci remain to be fully understood. Recent phylogenetic and evolutionary studies of *C. difficile* has been performed based on MLST, and groups of strains that are phylogenetically close to each other called ‘clades’ are now widely accepted, but the linkage of STs and clades with *agr* subtypes is not known to date.

The function of *agr* genes in *C. difficile* is also not well understood. Some previous studies showed that *C. difficile agr* genes positively enhance toxigenicity through a quorum-signalling substance supposed to have a thiolactone structure that the auto-inducer-peptides (AIPs) produced by *S. aureus agr* system also has [7–9]. Insertional deletion of *agrA* by the ClosTron system in the *agr2* locus of strain R20291 led to underexpression of flagellar biosynthesis genes in *C. difficile* [7].

The aim of this study was to investigate the epidemiology of *agr* genes in the context of a MLST-based phylogeny, laying the groundwork for future microbiological and clinical research of *C. difficile agr*.

## Methods

### *In silico* analysis of *C. difficile agr* loci on GenBank

Using genomic data of the *agr1* locus of *C. difficile* strain CD630 (GenBank accession number: AM180355.1) and *agr2R* locus of strain R20291 (GenBank accession number: FN545816.1) as template sequences [9], blast search for *agr* homologues was performed. In addition to previously known *agr* loci, the newly identified *agr2M* locus has the same four-component (*agrA*, *agrB*, *agrC*, *agrD*) structure as *agr2R* but with ~80 % nucleotide identity to *agr2R*. These three *agr* loci were targeted in the subsequent analysis by PCR.

### *C. difficile* isolates

A total of 133 non-duplicate *C. difficile* clinical isolates were obtained from the University of Tokyo Hospital between 2013 to 2014 [14]. Laboratory reference strains ATCC BAA-1382 and NCTC 13366 (hereafter referred to as CD630 and R20291, respectively) were also subjected to identical isolation methods and subsequent analysis.

### Genetic analysis of *agr* loci in *C. difficile* strains

**Fig. 1.**
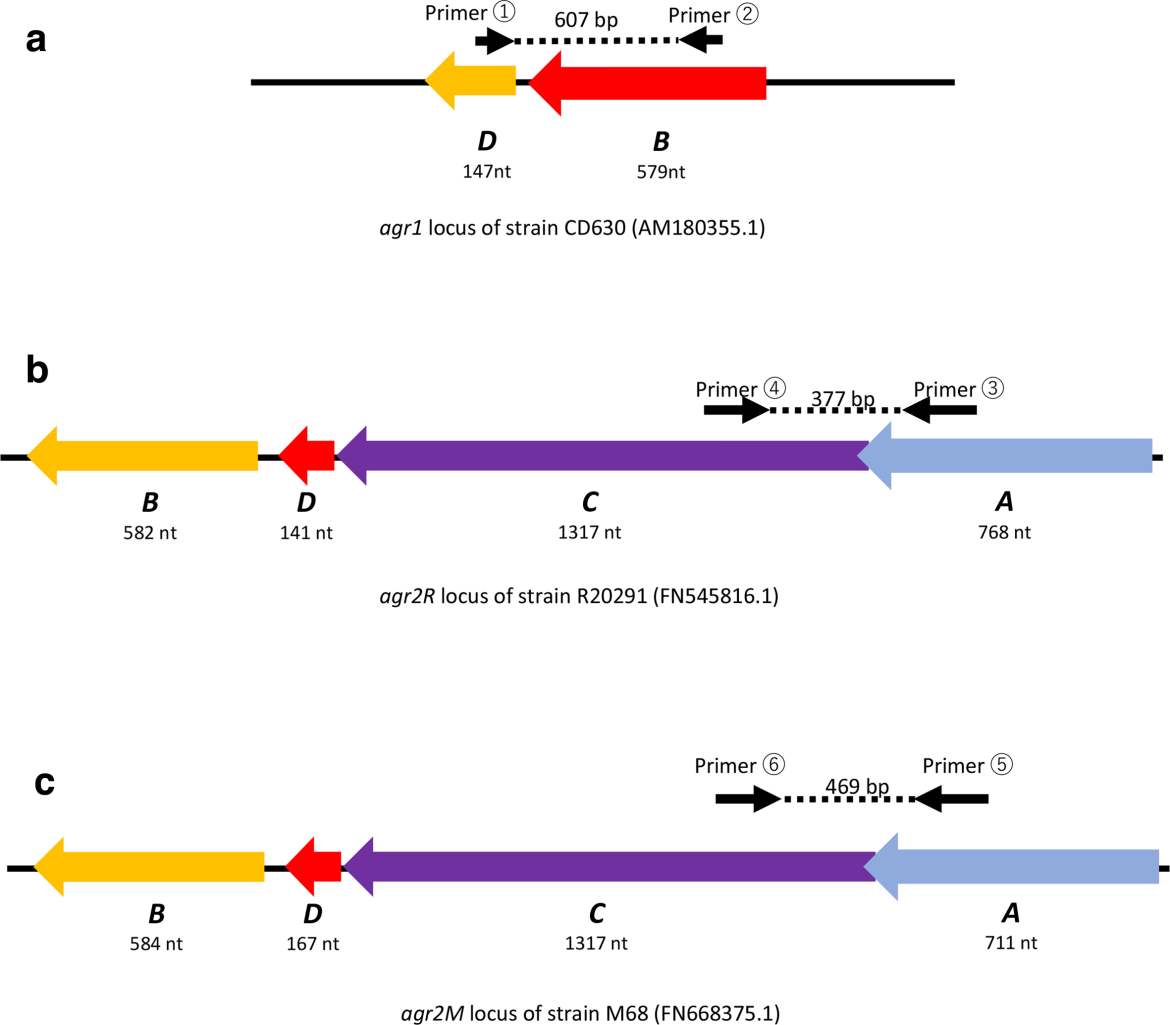
Genomic organization of (a) *agr1*, (b) *agr2R* and (c) *agr2M* loci. The targeted regions of the primer pairs in Table 1 are also shown, with the expected length of amplicons shown.

Both clinical and reference lab strains of *C. difficile* in our study were anaerobically cultured by our previous reported method [14]. *C. difficile* colonies on culture agar were inoculated into sterile water and then boiled at 95 °C for 10 min to make DNA templates for subsequent PCR using the Emerald Amp PCR Master Mix kit (Takara Bio, Shiga, Japan). A 100 bp DNA ladder (Toyobo, Osaka, Japan) was used to evaluate product size during gel electrophoresis of PCR amplicons. MLST and toxigenicity analysis were performed as described in previous reports [14–16]. As part of MLST analysis, PCR amplicons underwent DNA sequencing, and STs and clades were determined based on DNA sequencing data using the PubMLST sequence query page (https://pubmlst.org/cdifficile/). PubMLST was also used to determine STs and clades of some strains found in our *in silico* analysis. PCR of *agr* loci was performed on laboratory strains and clinical isolates targeting genetic regions overlapping with *agrB1* and *agrD1* of CD630(AM180355.1) using primer pairs agr1_BD_F/agr1_BD_R, *agr2R_C* and *agr2R_A* of R20291(FN545816.1) using pairs agr2R_AC_F/agr2R_AC_R, and *agr2M_C* and *agr2M_A* of M68(FN668375.1) using pairs agr2M_AC_F/agr2M_AC_R (Table 1, Fig. 1). Amplicons recovered from PCR underwent DNA sequencing by the Sanger method at Eurofins Genomics (Ebersberg, Germany) to confirm that the amplicons match the targeted genetic region. For samples negative for *agr1*-screening, additional PCR examination was performed to amplify the genomic region entirely, including *agr1*. Concatenated nucleotide sequences of housekeeper genes used to determine MLST was obtained from PubMLST website, and these datasets underwent phylogenetic analysis using PhyML via the Bio.Phylo library integrated with Biopython [17–19]. A phylogenetic tree was drawn using iTOL v5 (https://itol.embl.de/) [20], and clades of STs not specified on PubMLST were determined based on the tree.

**Table 1.** Primers used for screening of *agr1*, *agr2R* and *agr2M*

No.	Primer name	Sequence (5′-->3′)
1	agr1_BD_ F	GGCTGATGAATAATCCAAGGACAGGTACTA
2	agr1_BD_R	GCTTTCATAGTTAATATAACCACCATGC
3	agr2R_AC_F	GACCTACTGCAGAACCTTCAGC
4	agr2R_AC_R	GAGTTAAAGGCTTGAAACTTGC
5	agr2M_AC_ F	GTGAATTTGGATTTTTCAGATGCC
6	agr2M_AC_ R	AGCTAAACCTTCCCCCATC

## Results

### *In silico* analysis of agr subtypes among *C. difficile*

**Fig. 2.**
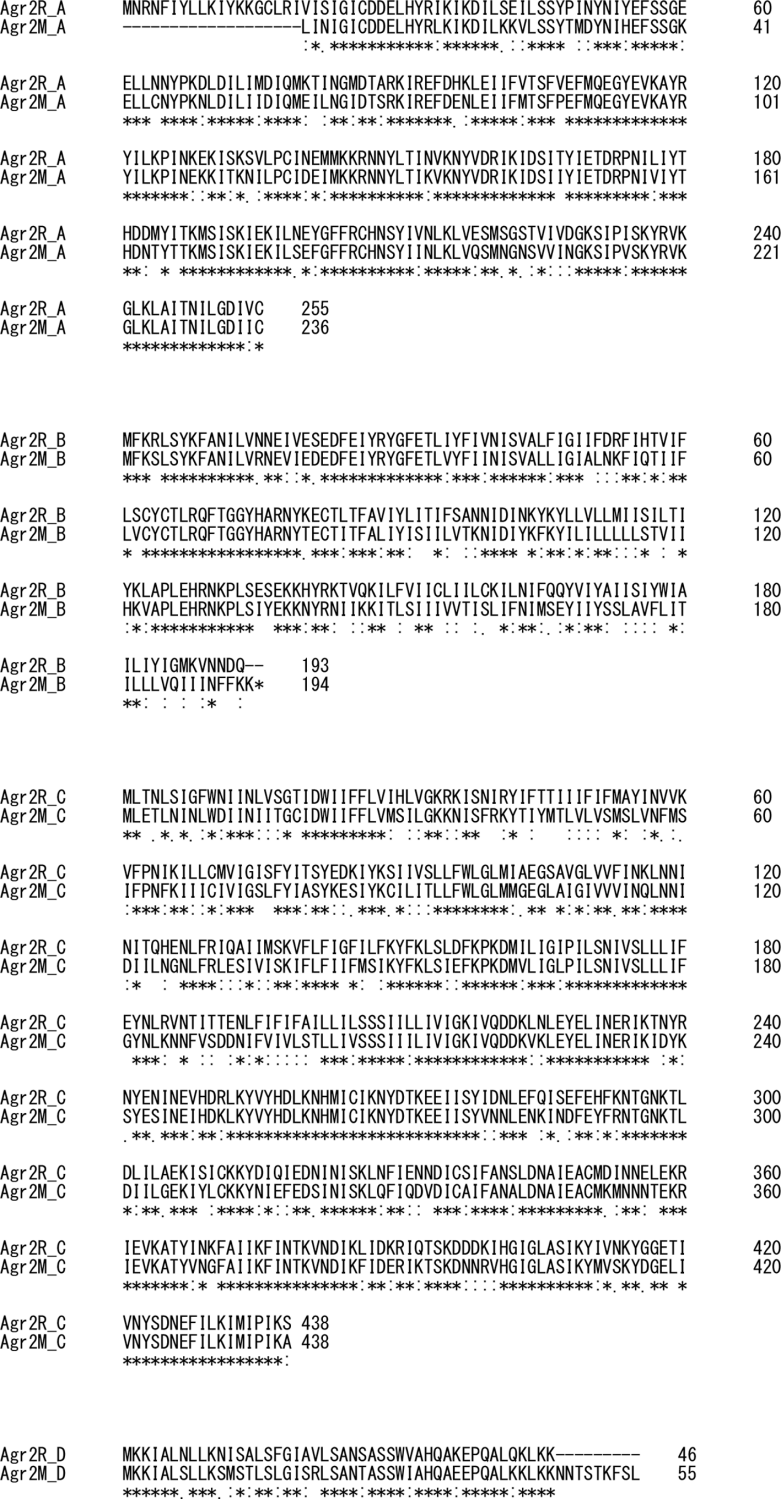
Multiple sequence alignment of *Agr2R* and *Agr2M* by Clustal Omega. Comparisons between amino acid sequences of *AgrB*, *AgrD*, *AgrC* and *AgrA* are shown. Symbols are as follows: an asterisk indicates identical, a colon means strongly similar, and a comma indicates weakly similar.

Using the *agr1* sequence of CD630 (GenBank accession number: AM180355.1) and the *agr2R* sequence of R20291 (GenBank accession number: AM545816.1) as templates, blast search was performed to find *agr* homologues using the genomic data of *C. difficile* strains registered at GenBank. Regarding *agr1*, almost all strains had genetic regions with over 96 % nucleotide-based identity to *agr1* of CD630. On the other hand, blast search using the nucleotide sequence of the R20291 *agr2* locus showed that, besides whole-genome sequences with genetic regions of over 95 % identity to *agr2R*, there were a group of strains that had genetic regions with around 80 % identity compared to the *agr2R* of strain R20291 (Table 2). One of these strains is M68 (GenBank accession number: FN668375.1), which is a toxin A-negative, toxin B-positive strain belonging to ST37/clade 4. To distinguish this from *agr2R*, the *agr2* locus in strains of M68 on GenBank was named *agr2M*. The genetic organization of the *agr* loci is shown in Fig. 1. Fig. 2 shows that the amino acid sequences predicted from ORF analysis of *agr2R* and *agr2M* shares many amino acids, but *AgrD* and *AgrA* show 10–20% difference in length between the two loci.

**Table 2.** List of whole-genome sequences (WGS) of *C. difficile* that likely possess *agr2M*. blast search showed that these WGSs have a region with 80.33–80.37 % nucleotide identity compared with *agr2R* region of R20291 (FN545816.1) and >95% identity compared with *agr2M* region of M68 (FN668375.1). Determination of ST/clade was performed using the PubMLST sequence query page

Strain	Accession No.	ST	Clade
DSM 29629	CP016104.1	ST39	Clade 4
DSM 29627	CP016102.1	ST37	Clade 4
CD161	CP029154.1	ST37	Clade 4
CDT4	CP029152.1	ST37	Clade 4
M68	FN668375.1	ST37	Clade 4
CF5	FN665652.1	ST86	Clade 4
DSM28669	CP012323.1	ST109	Clade 4
BJ08	CP003939.1	–*	–*
DSM29637	CP016106.1	ST83	Clade 1
CBA7204	CP029566.1	ST203	Clade 1

*WGS of BJ08 did not have *adk* (one of the seven housekeeper genes used in MLST), but the sequence of the other six housekeeper genes used to determine sequence type were identical to that of ST37.

### Epidemiology of *agr* genes in clinical isolates of *C. difficile*

**Fig. 3.**
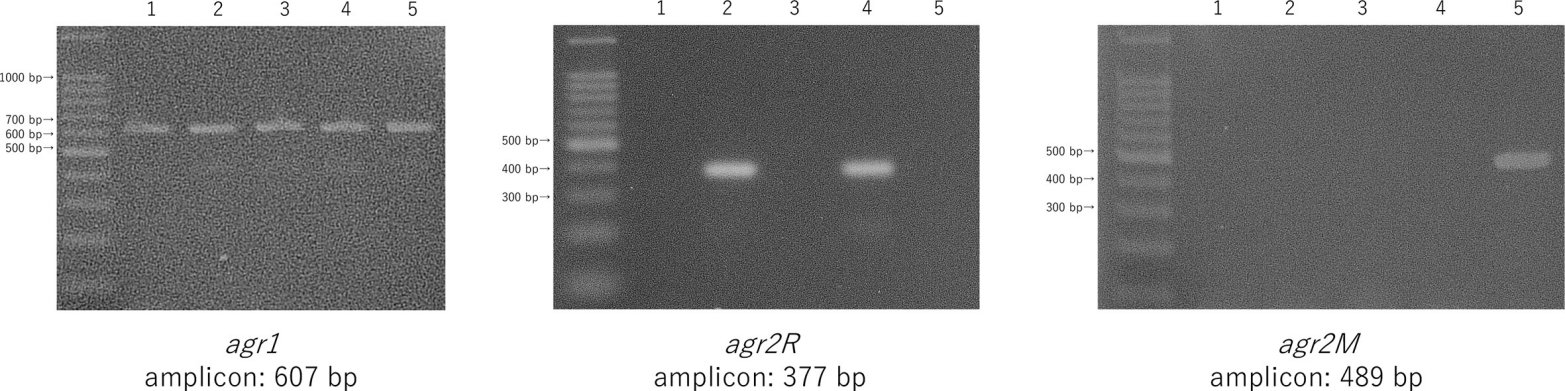
Visualization of PCR bands from *agr1*, *agr2R* and *agr2M* screening. 1: strain CD630 (laboratory strain); 2: strain R20291 (laboratory strain); 3: ST54/lade 1 (clinical isolate); 4: ST17/clade 1 (clinical isolate); and 5: ST81/clade 4 (clinical isolate).

PCR was performed on a total of 133 *C *. *difficile* isolates and two laboratory strains of CD630 and R20291 to confirm the presence of *agr1*, *agr2R* and *agr2M*. The position of the sequences corresponding to the primers is shown in Fig. 1. Fig. 3 shows examples of gel electrophoresis visualizing bands corresponding to 607, 377 and 469 bp amplicons yielded by PCR targeting *agr1*, *agr2R* and *agr2M*, respectively.

As of laboratory reference strains, PCR analysis confirmed that CD630 and R20291 both have a genetic region corresponding to *agr1*, but CD630 does not have *agr2R* or *agr2M*. In contrast, R20291 has the *agr2R* region. Of the total 133 clinical isolates, 131 isolates were positive for *agr1*, 61 isolates were positive for *agr2R*, and 26 isolates were positive for *agr2M*. The whole list of STs/clades, *agr* patterns and toxigenicity revealed in our study is shown in Table 3; the relationships between clades and *agr* patterns are summarized in Table 4.

**Table 3.** Sequence types, MLST-based clade category, toxigenicity (*tcdA* or *tcdB-positivity*), and *agr* status of isolated *C. difficile
*

Sequence types	Clade	No. of samples	*agr1*	*agr2R*	*agr2M*	toxigenicity
ST17	1	12	+	+	−	+
ST109	4	11	+	−	+	−
ST81	4	10	+	−	+	+
ST2	1	9	+	−	−	+
ST15	1	9	+	+	−	−
ST54	1	9	+	−	−	+
ST8	1	8	+	+	−	+
ST3	1	5	+	+ (*n*=4) − (*n*=1)	−	+ (*n*=4) − (*n*=1)
ST37	4	4	+	−	−	+
ST100	1	4	+	+	−	−
ST26	1	3	+	−	−	−
ST35	1	3	+	+	−	+
ST53	1	3	+	+	−	+
ST11	5	2	+	+ (*n*=1) − (*n*=1)	−	+
ST14	1	2	+	−	−	+
ST48	1	2	+	+	−	+ (*n*=1) − (*n*=1)
ST55	1	2	+	−	−	+
ST401	4	2	+	−	−	−
ST407	C-Ⅰ	2	+	−	−	−
ST5	3	1	+	−	−	+
ST28	1	1	+	−	−	−
ST41	2	1	+	+	−	+
ST42	1	1	+	+	−	+
ST49	1	1	+	−	−	+
ST58	1	1	+	−	−	+
ST63	1	1	+	+	−	+
ST66	1	1	+	+	−	+
ST123	2	1	+	+	−	+
ST129	1	1	+	+	−	+
ST153	1	1	+	+	−	+
ST159	4	1	+	−	+	−
ST183	1	1	+	−	−	+
ST198	4	1	+	+	−	+
ST201	3	1	+	−	−	+
ST205	1	1	+	+	−	−
ST223	2	1	+	+	−	+
ST243	4	1	+	−	−	−
ST247	1	1	+	+	−	+
ST278	1	1	+	−	−	+
ST297	C-Ⅰ	1	−	−	−	−
ST301	1	1	+	+	−	+
ST303	C-Ⅰ	1	+	−	−	−
ST304	1	1	+	−	−	+
ST400	1	1	+	+	−	+
ST402	C-Ⅲ	1	−	−	−	−
ST403	4	1	+	−	−	−
ST404	1	1	+	+	−	+
ST405	4	1	+	−	−	−
ST406	1	1	+	+	−	−
ST408	1	1	+	−	−	+

**Table 4.** Relationship between *agr* patterns and clades

Clade	1	2	3	4	5	C-Ⅰ	C-Ⅲ	total
*agr1*	32	0	2	5	1	3	0	44
*agr1 +agr2R*	57	3	0	1	1	0	0	61
*agr1 +agr2M*	0	0	0	26	0	0	0	26
Negative	0	0	0	0	0	1	1	2
Total	89	3	2	32	2	4	1	133

Some STs showed peculiar patterns of *agr*, such as *agr1*-negative ST297 and ST402, as well as *agr2R*-positive ST198 belonging to clade 4. As most STs contained only a single pattern of *agr* loci, ST3 and ST11 were characteristic in that both *agr2R*-positive and *agr2R*-negative strains belonged to them.

### Phylogenetic analysis based on STs from our clinical isolates and STs representing known clades

**Fig. 4.**
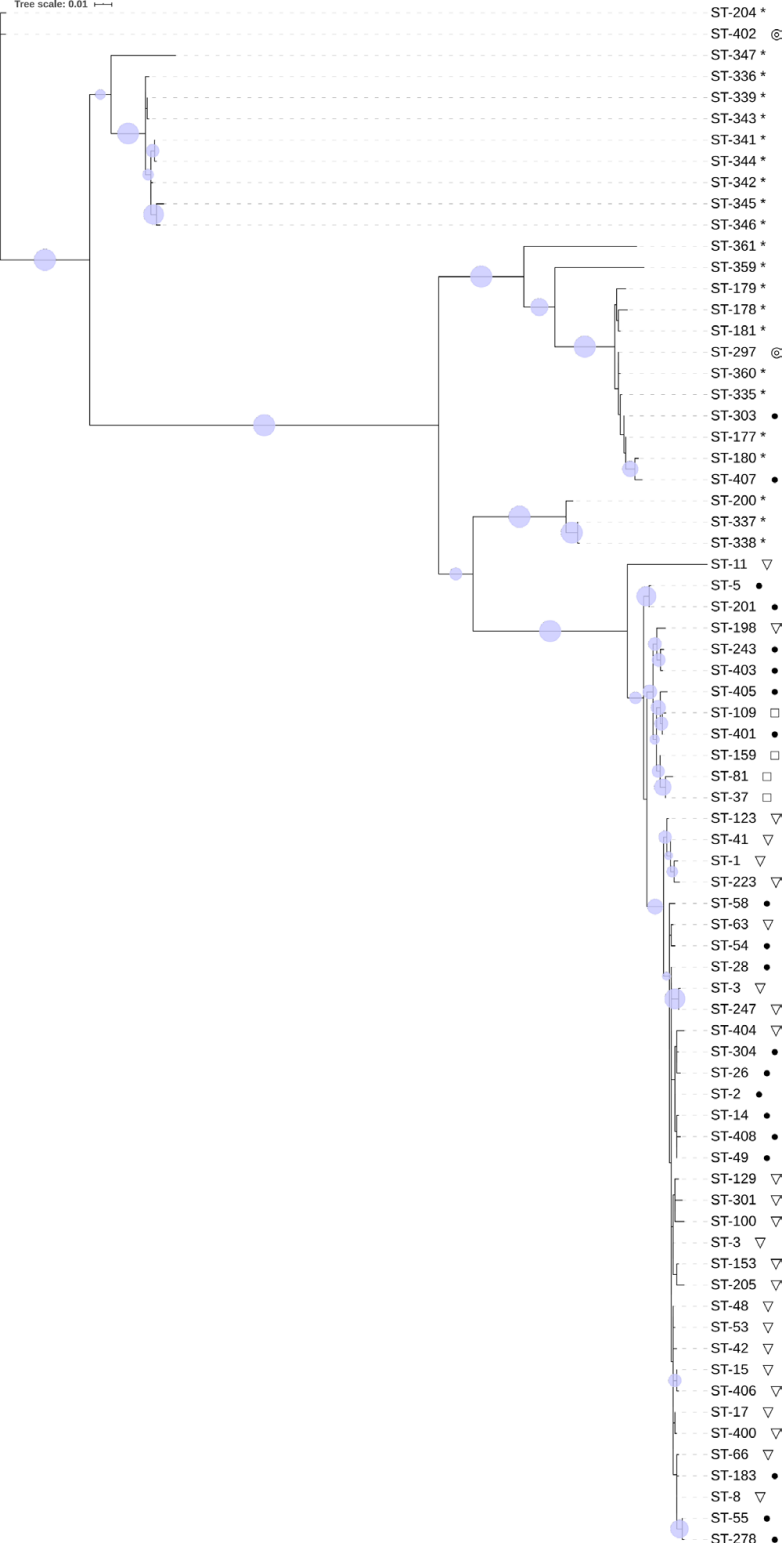
Maximum-likelihood phylogenetic tree (model TN93, 1000 replicates) based on analysis by PhyML. STs found in our clinical samples; ST1 (corresponding to R20291 strain) and STs used in previous studies as references for clade C-Ⅰ, C-Ⅱ and C-Ⅲ underwent this analysis. Circles are drawn on branches with bootstrap value over 50. ●: *agr1*; ▽: *agr1 +22R*; □: *agr1 +22M* ; ◎: no *agr* locus; *: reference strains.

STs found in this study underwent MLST-based phylogenetic analysis together with ST1 (clade 2) and STs previously reported to be of clade C-Ⅰ, C-Ⅱ and C-Ⅲ [21–26]. Phylogenetic analysis was performed using the maximum-likelihood method with the concatenated sequences of seven housekeeper genes, applying the MLST method. The resulting phylogenetic tree is shown in Fig. 4.

In clade 1, no branching point with a bootstrap value over 70 was shown to be a border between STs with *agr1* and STs with *agr1+agr2R*, suggesting that in terms of bootstrap value, these two groups are not clearly demarcated from each other. This can also be said of clade 4, as the tree shows no branching point dividing STs with *agr1+agr2M*, *agr1+agr2R* and *agr1* patterns.

The phylogenetic tree also shows that ST297 (previously reported to be a ST of clade C-Ⅰ [22]) belongs to a group of neighbours in clade C-Ⅰ. As mentioned above, the tree shows that ST303 and ST407 were confirmed to have *agr1* and also belong to clade C-Ⅰ. The closest ST to ST402 in the phylogenetic tree is ST204, which was previously mentioned to be clade C-Ⅲ [26]; other STs of clade C-Ⅲ are also relatively close to ST402.

## Discussion

This is the first study focusing on a phylogenetic analysis of the *agr* locus in clinical isolates of *C. difficile *. Based on our finding that two relatively different subtypes of *agr* loci exists in *agr2*, the epidemiology of *agr* in clinical isolates of *C. difficile* was elucidated. Previous studies on *C. difficile agr* mainly focused on regulatory effects of *agr* genes on virulence factors, such as toxin production in laboratory strains [7–9], so the finding that not only toxigenic strains but also nontoxigenic strains possess these genes is of note and warrants further analysis of unknown functions for the *agr* loci of *C. difficile *. Potential new functions of *agr2M* are also of interest, and we are the first to report this locus.

Our study not only adds to previous reports showing the universality of *agr1* and the distribution of *agr2R* among clinical and laboratory *C. difficile* isolates [13, 27] but also revealed the distribution of *agr2M*. The discrepancy of *agr* patterns within a single ST observed in some STs may provide clues to help uncover the evolutionary pathway of *C. difficile *.

A previous study by Darkoh *et al*. [9] showed that examined laboratory strains of *C. difficile* could live without the *agr1* locus. The presence of *agr1*-negative isolates in our study also suggests that loss of *agr1* in *C. difficile* is not lethal, and the same thing can be said about *agr2R* and *agr2M*.

The subject of our phylogenetic analysis does not represent the whole population of *C. difficile *, but still, several notable findings about *agr* were obtained. First, a deviation of *agr* patterns in some local parts of the phylogenetic tree/clades was observed. For example, almost all *agr2R*-positive strains were in clade 1 or 2, and all STs with *agr2M* were in clade 4. Consistent with these results, a previous phylogenetic study of *C. difficile* suggests that *agr2M* is mainly found in clade 4 and rarely in clade 1 [28]. Interestingly, the subclade-like group in clade 4, which the *agr2R*-positive ST198 belongs to, includes two *agr2R*-negative STs without *agr2M*, whereas another subclade in clade 4, including ST37 and ST81, does not have any STs with *agr2R*.

The fact that single *agr1*-negative isolates were found in both clade C-Ⅰ and clade C-Ⅲ suggests that loss of *agr1* may have occurred at some point during their evolution. The validity of bacterial identification of clade C-Ⅰ strains was questioned in a previous report based on the fact that the average nucleotide identity score between strains of clade C-Ⅰ and clade 1–5 were low [22]. Not only genetic but also functional characterization of *C. difficile* clades is expected to provide further insight into bacteriological evolution and taxonomy, and analysis of *agr* function may play a key role in moving the field forward.

In summary, our study sheds light on the previously unknown genetic diversity and molecular epidemiology of the *agr* loci in *C. difficile *. Together with our MLST-based phylogenic analysis, our findings may offer novel insights into the *agr* loci, leading to further functional, evolutionary and clinical research of this gene.
